# Noninvasive Prenatal Testing of Methylmalonic Acidemia cblC Type Using the cSMART Assay for *MMACHC* Gene Mutations

**DOI:** 10.3389/fgene.2021.750719

**Published:** 2022-01-07

**Authors:** Weigang Lv, Lili Liang, Xin Chen, Zhuo Li, Desheng Liang, Huimin Zhu, Yanling Teng, Weijuan Wu, Lingqian Wu, Lianshu Han

**Affiliations:** ^1^ Center for Medical Genetics and Hunan Key Laboratory of Medical Genetics, School of Life Sciences, Central South University, Changsha, China; ^2^ Department of Pediatric Endocrinology and Genetic Metabolism, Xinhua Hospital, Shanghai Institute of Pediatric Research, School of Medicine, Shanghai Jiao Tong University, Shanghai, China; ^3^ Hunan Jiahui Genetics Hospital, Changsha, Hunan, China

**Keywords:** noninvasive prenatal testing, methylmalonic acidemia cblC type, circulating single molecule amplification and re-sequencing technology, monogenic disorder, next-generation sequencing, cell-free DNA

## Abstract

Noninvasive prenatal testing (NIPT) for monogenic disorders has been developed in recent years; however, there are still significant technical and analytical challenges for clinical use. The clinical feasibility of NIPT for methylmalonic acidemia cblC type (cblC type MMA) was investigated using our circulating single-molecule amplification and re-sequencing technology (cSMART). Trios molecular diagnosis was performed in 29 cblC type MMA-affected children and their parents by traditional Sanger sequencing. In the second pregnancy, invasive prenatal diagnosis (IPD) of the pathogenic *MMACHC* gene was used to determine fetal genotypes, and NIPT was performed using a novel *MMACHC* gene–specific cSMART assay. Maternal–fetal genotypes were deduced based on the mutation ratio in maternal plasma DNA. Concordance of fetal genotypes between IPD and NIPT, and the sensitivity and specificity of NIPT were determined. After removing two cases with a low *P* value or reads, the concordance ratio for NIPT and IPD was 100.00% (27/27), and the sensitivity and specificity were 100.00% (54.07–100.00%) and 100.00% (83.89–100.00%), respectively. This study demonstrates that NIPT using the cSMART assay for cblC type MMA was accurate in detecting fetal genotypes. cSMART has a potential clinical application as a prenatal diagnosis and screening tool for carrier and low-risk genotypes of cblC type MMA and other monogenic diseases.

## Introduction

Methylmalonic acidemia (MMA) is an autosomal recessive metabolic genetic disorder. Cobalamin C type MMA (cblC type MMA, MIM #277,400) is the most common form of MMA in China. The incidence of cblC type MMA ranges from 1:60,000 to 1:100,000 in the United States (Cusmano-Ozog et al., 2007; Weisfeld-Adams et al., 2010) and 1:3,920 to 1:21,488 in China (Han et al., 2016; Guo et al., 2018; Zhou et al., 2018). Patients with cblC type MMA exhibit a wide spectrum of clinical manifestations and usually exhibit multisystem abnormalities with varying degrees of severity (Weisfeld-Adams et al., 2013; Fischer et al., 2014). More than 90% of cblC cases are serious during the neonatal period, with the most severe consequences being abnormalities and stillbirth (Rosenblatt et al., 1997; Fischer et al., 2014).

The gene responsible for cblC type MMA is *MMACHC* (MIM *609,831), which maps to chromosome region 1p34.1 and consists of four coding and one non-coding exon. More than 100 pathogenic mutations have been identified in patients with cblC type MMA disease (Hu et al., 2018; Wang et al., 2019). The mutation spectrum of *MMACHC* varies greatly among populations. In the Chinese population, the five most common *MMACHC* mutations were c.609G > A (p.Trp203Ter), c.658_660delAAG (p.Lys220del), c.80A > G (p.Gln27Arg), c.482G > A (p.Arg161Gln), and c.394C > T (p.Arg132Ter), and they account for more than 75% of all pathogenic variants (Liu et al., 2010; Hu et al., 2018; Wang et al., 2019).

An increasing number of cblC type MMA patients have been found to exhibit better outcomes with an early diagnosis through expanded newborn screening, particularly by liquid chromatography tandem mass spectrometry (LC-MS/MS) (Oglesbee et al., 2008). Despite the reduced mortality and morbidity in cblC-affected children, their quality of life remains very low, with great economic burden on their families. One of the most effective interventions is to avoid the birth of children with cblC type MMA by prenatal diagnosis, which is commonly performed using genetic testing and biochemical measurement of amniotic cells/fluid (Zhang et al., 2008; Inoue and Ohse, 2011; Ji et al., 2019).

The discovery of cell-free fetal DNA (cfDNA) has enabled noninvasive prenatal testing (NIPT) for many genetic disorders, and NIPT for monogenic disorders has become a rapidly developing field (Lo et al., 1997). However, there are still significant technical and analytical challenges in the use of NIPT. Circulating single-molecule amplification and re-sequencing technology (cSMART), which is reliable for NIPT of several monogenic disorders (Lv et al., 2015; Chen et al., 2016; Han et al., 2017; Duan et al., 2018; Lv et al., 2019; Lv et al., 2021) was previously adapted. In this study, the targeting primers employed by this technique were expanded and redesigned to cover pathogenic mutations in *MMACHC*. The advanced maximum likelihood estimation (MLE) was introduced to deduce maternal and fetal genotypes. This customized cSMART assay was applied to maternal plasma samples from 29 pregnant women at risk for cblC type MMA disease and evaluated its performance by comparing it with that of the gold-standard, invasive prenatal diagnosis (IPD), using fetal amniocytes.

## Materials and Methods

### Study Design

**FIGURE 1:**
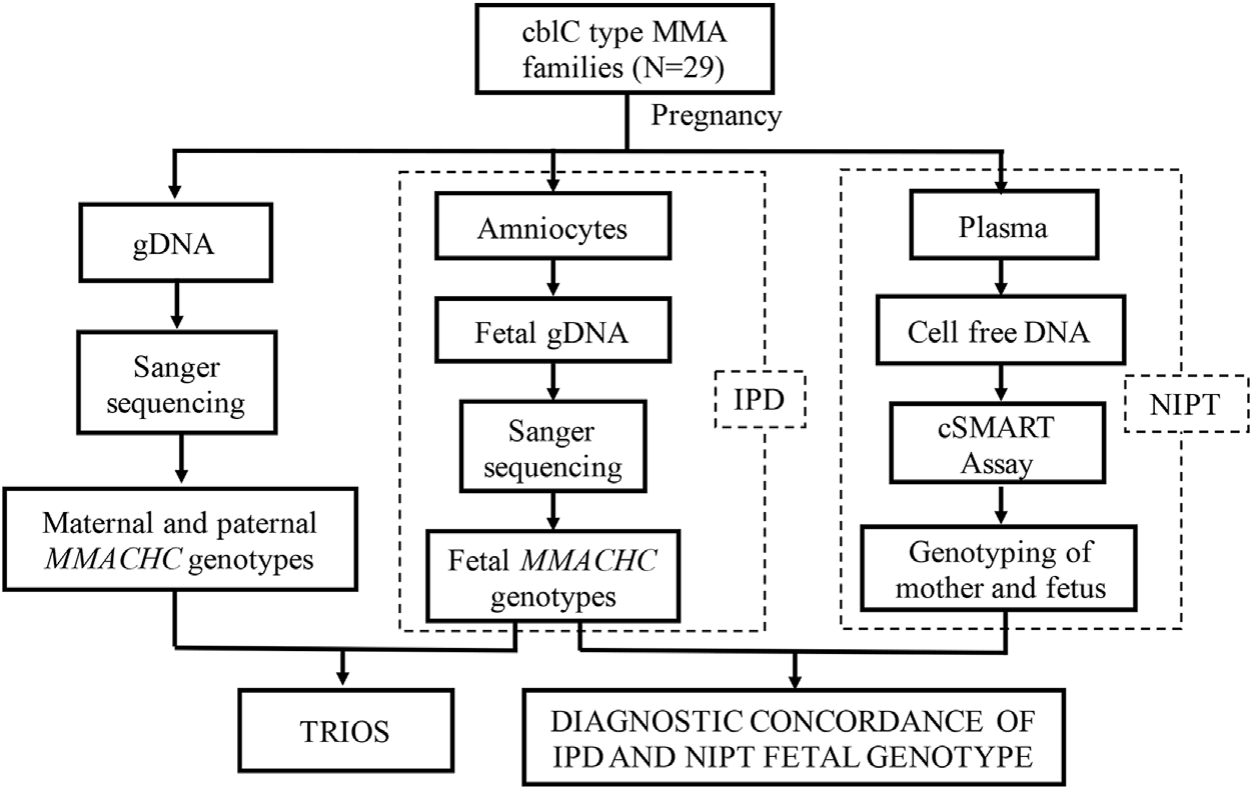
Study design. Trio gene testing to identify pathogenic *MMACHC* mutations. IPD and NIPT were performed to assess fetal genotypes in second pregnancies.

29 families with cblC type MMA disease diagnosed during their first pregnancy were recruited from Shanghai Xinhua Hospital and Hunan Jiahui Genetic Hospital. After receiving genetic counseling, all families agreed to be detected for pathogenic mutations in the *MMACHC* of the probands and parents. No abnormality was found for these couples in physical examination. Traditional IPD was provided for the next pregnancy, in which the family’s pathogenic mutations were detected using fetal genomic material obtained through amniocentesis. Meanwhile, plasma of the pregnant woman was collected, and NIPT was performed using cSMART (Figure 1). The gestational week and maternal age were recorded. All the research processes were approved by the Medical Ethics Committee of Xin Hua Hospital affiliated to Shanghai Jiao Tong University School of Medicine (approval no. XHEC-D-2014-026, December 8, 2014) and Hunan Jiahui Genetic Hospital (approval no. 2014121501, December 15, 2014), and written informed consent was obtained from all of the recruited subjects. Patients were not involved in the development of the research, and there were no core outcome sets used in this study.

### Family DNA Testing and Invasive Prenatal Diagnosis

DNA testing of each trio was initially undertaken using exon PCR and Sanger sequencing to identify pathogenic *MMACHC* mutations of proband and inheritance from parents. Clinical significance of each variant identified was assessed following the ACMG standards and guidelines (Richards et al., 2015). For prenatal diagnosis, fetal cells were retrieved by amniocentesis, and genomic DNA was tested for the parental pathogenic mutations using PCR and Sanger sequencing.

### cSMART Assay for the Detection of *MMACHC* Mutations in Maternal Plasma

**FIGURE 2:**
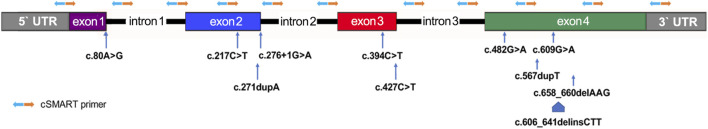
Distribution of primers and known mutant alleles tested for the *MMACHC* gene in this study. Primer pairs for the inverse targeting PCR step of the cSMART assay are denoted by orange and blue arrows.

Before amniocentesis, 10 ml peripheral blood of each pregnant woman was collected in a Streck BCT tube (Streck, Omaha, NE, USA) and transported to an NIPT lab at room temperature (25°C) within 2 days. About 5 ml plasma was separated by two rounds of centrifugation. cfDNA was extracted by QIAamp Circulating Nucleic Acid Kit (Qiagen, Hilden, Germany) from about 2.5 ml plasma according to the manufacturer’s instructions. The cSMART assay was carried out as previously described: 11 newly designed targeting primers (Supplementary Table 1) were covering the four coding exons of *MMACHC* (Figure 2), and 76 reserved heterozygous SNP primers (Supplementary Table 2) were used for calculating the fetal DNA fraction (FF) (Song et al., 2016; Lv et al., 2019; Lv et al., 2021). Library preparation for the cSMART assay was performed, including end ligation with the barcoding adapter, PCR amplification, cyclization, inverse targeting PCR, and amplification using primers with sequencing and case index sequences as described in previous studies (Lv et al., 2019; Lv et al., 2021). In detail, end ligation of cfDNA was performed with degenerative seven base barcode adapters using T4 DNA Ligase (Rapid) (Enzymatics, Beverly, MA, USA) at 20°C for 15 min and at 65°C for 10 min, and a 15-cycle PCR amplification was performed using NEBNext® Ultra™Ⅱ Q5® Master Mix (NEB, Ipswich, MA, USA). Amplification product was circularized by complementary bridge oligos using Taq DNA ligase (NEB, Ipswich, MA, USA). The library was divided into two equal sub-libraries, and using NEBNext® Ultra™Ⅱ Q5® Master Mix (NEB, Ipswich, MA, USA) for 25 cycles, inverse PCR was targeted by two different multiplex primer sets as listed in Supplementary Table 1. The final library was generated by PCR amplification using Phusion PCR Master Mix (NEB, Ipswich, MA, USA) for 15 cycles. The final cSMART library was mixed and sequenced on an Illumina MiSeq platform (Illumina, San Diego, CA, USA) in the 250 bp × 2 paired-end mode.

### Data Analysis and Fetal Genotyping of NIPT

The sequencing data of the cSMART library were processed, as previously described (Lv et al., 2019; Lv et al., 2021). Reads amplified using primers across targeted alleles were deleted, and reads containing the highest frequency of SNPs were retained to obtain a consistent sequence (unique read). *MMACHC* mutation alleles were determined by calculating the ratio of mutants to the total unique reads in plasma DNA. Fetal DNA fractions were calculated based on the allelic fractions of the 76 selected heterozygous SNPs as described in Supplementary Material 2 (Song et al., 2016). The fetal allelic fraction (Δ) is calculated to half of the FF. Δ, and mutant and total unique reads were integrated to account for the most likely maternal–fetal genotypes using the MLE (Jiang et al., 2012; Lv et al., 2019; Lv et al., 2021).

The maximum likelihood estimation (MLE) based on binomial distribution was used to deduce the maternal–fetal genotypes and *P* value detailed in Supplementary Material 3. A formula based on MLE utilizes the observed mutant unique reads (m), Δ (%, to deduce ε), the total unique read (T), and the most likely genotype (G) and the corresponding probability (*P(G)*) to be deduced as follows:

$$P\;\left(G\right)=\;\frac{max\left(\left(\begin{array}{c} T\\ m \end{array}\right)\varepsilon_{k}^{m}{\left(1-\varepsilon_{k}\right)}^{T-m}\right)}{\sum_{G_{k}}\left(\begin{array}{c} T\\ m \end{array}\right)\varepsilon_{k}^{m}{\left(1-\varepsilon_{k}\right)}^{T-m}},$$

where k∈(AA/AA,AA/Aa,Aa/AA,Aa/Aa, aa/aa), and the expected values of $\varepsilon_{AA/AA},\;\varepsilon_{AA/Aa},\;\varepsilon_{Aa/AA},\;\varepsilon_{Aa/Aa},\text{ and }\varepsilon_{aa/aa}$ are 0, Δ, 0.5-Δ, 0.5, and 0.5+Δ, respectively. AA/Aa represents the maternal genotype AA and the fetal genotype Aa, and the Aa/aa genotype was regarded as a mirror of Aa/AA.

In the description of maternal–fetal genotypes deduced using the MLE, major (maternal) alleles detected in the cfDNA were denoted with the capital letters “A” (wild type) and “B” (mutant), while minor (fetal) alleles were denoted with the small letters “a” (wild type) and “b” (mutant) (Table 1). The cutoff for quality control (QC) to call a valid maternal–fetal genotype was set as FF ≥ 5%, total sequencing reads ≥500, and P value ≥70%, according to our previous study (Lv et al., 2021).

**TABLE 1:** Genotype description deduced from the maternal–fetal genotype for the inherited maternal and paternal alleles.

Carrier couples	Fetal genotype	Inherited from maternal allele*	Inherited from paternal allele*	Concordance of NIPT genotype with IPD (by QC)			
				Genotypes, n (%)	Concordant, n (%)	Discordant, n (%)	Concordance rate (%)
Heterozygote	Normal homozygote	ABaa	AAaa**	2 (2)	2 (2)	0 (0)	100.00% (100.00%)
	Maternal heterozygote	ABab	AAaa**	7 (7)	7 (7)	0 (0)	100.00% (100.00%)
	Paternal heterozygote	ABaa	AAab	9 (9)	9 (9)	0 (0)	100.00% (100.00%)
	Compound heterozygote	ABab	AAab	5 (5)	5 (5)	1 (1)	100.00% (100.00%)
		Inherited from maternal (paternal) allele*					
Homozygote	Normal homozygote	ABaa		2 (2)	2 (2)	0 (0)	100.00% (100.00%)
	Carrier heterozygote	ABab		3 (1)	2 (1)	1 (0)	66.67% (100.00%)
	Affected homozygote	ABbb		1 (1)	1 (1)	0 (0)	100.00% (100.00%)
	All genotypes			29 (27)	28 (27)	1 (0)	96.55% (100.00%)

Maternal alleles detected in the cfDNA are indicated by the symbols A (wild type) and B (mutant), while fetal alleles are indicated by the symbols a (wild type) and b (mutant). * The parent alleles were unknown in this NIPT study; thus, genotypes that inherited no paternal allele yielded no result and have been assigned the symbol AAaa** in the table for uniform representation.

## Results

### Genetic Testing for Probands and Their Parents

29 probands were identified as cblC type MMA-affected, with pathogenic variants of the *MMACHC* gene, and the variants derived from their parents were validated by Sanger sequencing (Supplementary Table 3). Eleven types of mutations were found in probands with *MMACHC* mutant hotspots, and the most common were c.609G > A (29/58, 50.00%), c.658_660delAAG (12/58, 20.69%), c.217C > T (3/58, 5.17%), c.482G > A (3/58, 5.17%), and c.567dupT (3/58, 5.17%), followed by c.80A > G (2/58, 3.45%), c.394C > T (2/58, 3.45%), c.271dupA (1/58, 1.72%), c.276+1G > A (1/58, 1.72%), c.427C > T (1/58, 1.72%), and c.606_641delinsCTT (1/58, 1.72%). There were six homozygotes (five for c.609G > A and one for c.567dupT) with the same allelic mutation and 23 compound heterozygotes with two different compound allelic mutations among the 29 cases studied.

### Traditional Invasive Prenatal Diagnosis for cblC Type MMA

Recruited couples agreed to IPD when they conceived a singleton pregnancy naturally. Gene testing of pathogenic alleles of *MMACHC* was performed for all the 29 pregnancies in the second trimester (Supplementary Table 3). There were 17 carriers of such alleles (eight maternal and nine paternal heterozygotes), eight individuals who were affected by them (three homozygotes and five compound heterozygotes), and four individuals with the wild-type alleles (normal homozygotes).

### Performance of NIPT in Fetal *MMACHC* Genotyping

The NIPT assay was performed using coded maternal plasma DNA in an independent NIPT laboratory. For each NIPT result, the FF and mutation ratio were first generated using a bioinformatics pipeline; then, maternal–fetal genotypes were deduced with the corresponding probability value (*P*) (Supplementary Table 3). The mean total allelic template number was 902 ± 293 (range 486–1754), which was calculated by counting unique reads with the same barcode and genomic position. The mean value of the fetal fraction was 11.07 ± 2.18% (range 8.63–17.37%), which was determined using informative heterozygous SNPs. Based on the high allelic templates and FF, fetal genotypes were assigned with high confidence. Overall, 79.31% (23/29) of maternal allele mutations and 100.00% (14/14) of paternal allele mutations had a *P* value >90% to deduce fetal–maternal genotype from maternal cfDNA, further demonstrating the high performance of the assay developed for genotype assignment.

**FIGURE 3:**
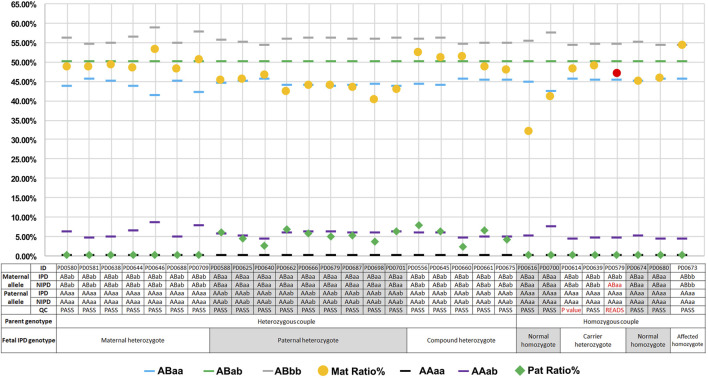
Plots of mutation percentages in the plasma DNA and maternal–fetal genotypes of 29 cases. Blue (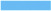), green (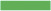), gray (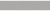), black (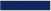), and purple (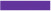) rectangles denote theoretical mutation deviation for genotypes of ABaa, ABab, ABbb, AAaa, and AAab deduced using half of FF, respectively. Orange circles (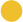) and green diamonds (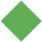) denote observed maternal and paternal mutation percentages in cfDNA, respectively, while discordant maternal is indicated by a red circle (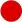). The table below shows deduced maternal–fetal genotype and the corresponding number of each case. Discordant and failed QC value cases are indicated by red color. IPD, invasive prenatal diagnosis; NIPT, noninvasive prenatal testing; Mat, maternal; Pat, paternal.

Upon comparing NIPT with IPD, the original concordance ratio was found to be 96.55% (28/29) (Figure 3). After applying the set cutoff values mentioned above, two QC-failed cases were removed, and the concordance ratio was found to be 100.00% (27/27). For the 27 QC-passed NIPT cases, there were no false-negative or false-positive results, and the sensitivity and specificity for fetal genotyping were 100.00% (54.07–100.00%) and 100.00% (83.89–100.00%), respectively.

## Discussion

### Main Findings

The cSMART assay was applied to cover the entire coding sequence in the exons and the proximal flanking intron regions of the *MMACHC* gene. Blinded examination performed in 29 cblC type MMA high-risk couples demonstrated that NIPT was 96.55% (28/29) concordant with IPD. The concordance rate reached 100.00% in 27 cases after two cases were removed based on the QC criteria. No false-positive or false-negative result was observed in the following clinical pathogenicity assessment. The results revealed a sensitivity of 100.00% (54.07–100.00%) and a specificity of 100.00% (83.89–100.00%), which suggested a high performance of our assay in future diagnostic applications of cSMART.

### Strengths and Limitations

This is the first report of the clinical application of a direct sequencing method, unlike the haplotype-based indirect methods, in NIPT for cblC type MMA (Han et al., 2020). It is proven again that the cSMART assay can be easily customized to target exonic coding sequences and the exon proximal intronic regions of interesting genes (Lv et al., 2019). After removing the double of primer size (average about 21bp) from the size of cfDNA (average about 166bp) (Lo et al., 2010), the maximum length of covering sequences by primers was about 124bp (Figure 2), and the coverage can be doubled using upstream and downstream primer pairs. Moreover, primers were in average about 55bp from the exon–intron boundaries, and cSMART can cover about 179bp (55bp + 166bp-21bp × 2) exon proximal flanking intronic regions maximally (Supplementary Figure 1).

Strengthen quality control was performed to reduce contamination between samples during operation, such as double-checking in each experiment step. Moreover, before plasma separation, different non-human homologous, artificial barcoded DNA fragments are mixed and recorded for different samples, and the comparison of the barcoded DNA after data analysis can be used to judge whether there is contamination between the samples.

The current cSMART assay is not designed for the detection of genomic copy number variation (CNV) and exon(s) deletion/duplication. For example, to detect deletions of the α-thalassemia gene at the level of kilobases using cSMART, it requires careful designing of a great number of effective primers as well as the corresponding mutation dosage algorithm (Lun et al., 2008). The same principle applies for diseases caused by exon(s) deletion/duplication, such as Duchenne muscular dystrophy. Unfortunately, manual design and optimization of cSMART primers are very time-consuming, although it can be improved using the machine learning algorithm. Besides, the detection efficiency of nucleotide small insertion or deletion (indels) is not as good as nucleotide substitutions for the cSMART assay, and alternative alignment and SNP calling algorithm should be chosen for testing of indels.

MMA can be classified as isolated methymalonic acidemia and combined methymalonic acidemia with homocystinuria. The cblC type chosen in this study accounted for 94.7% of Chinese MMA combined with homocystinuria (Liu et al., 2018) but not for the other four subtypes of the combined cobalamin metabolism (cblD, cblF, cblJ, and cblX) in our current assay (Yu et al., 2013; Richard et al., 2018). However, this can be easily addressed using appropriate additional targeting primers. For cblC type MMA, eleven common alleles were identified, including the top five common mutations in the Chinese population (c.609G > A, c.658_660delAAG, c.80A > G, c.482G > A, and c.394C > T) (Liu et al., 2010; Hu et al., 2018; Wang et al., 2019). NIPT primers also covered the most common variants in the Caucasian population, c.271dupA (48.05%), c.394C > T (13.65%), and c.331C > T (7.36%) (Wang et al., 2019); however, international cooperation is required to evaluate it.

### Interpretation

For the two QC-failed cases (PD0614 and PD0579), blood collection, sample transportation, and extraction processes were standard; DNA quantity and gestational age were also close to the average. The first testing of these two cases was with low *P* or sequencing reads below QC (*p* = 58.12% for PD0614; *p* = 66.96% and read number = 486 for PD0579), and the cSMART assay was repeated using plasma DNA (Supplementary Table 4). Values were still lower than the cutoff value (*p* = 57.9% for PD0614; *p* = 77.33% and read number = 429 for PD0579), suggesting that discordance did not occur due to the experimental operation, but may be due to the sample itself.

In traditional newborn screening, high C3 and C3/C2 ratios are signs of the abnormal metabolism of propionate due to cblC type MMA (Weisfeld-Adams et al., 2010; Huemer et al., 2015). However, traditional newborn screening is not popular in undeveloped areas for above metabolic diseases, and newborn genetic screening is not prevalent worldwide, and there is no large-scale genetic screening of carrier couples for metabolic diseases. Once the advanced version of cSMART for common inborn error of metabolism reaches high performance as IPD, direct selection of NIPT can be used to determine both maternal carrier genotype and fetal parent-derived genotype for low-risk populations. The assay cost is equivalent to that of traditional molecular assays (Lv et al., 2019), and NIPT saves the cost of invasive operation and preoperative examination for IPD, and provides guidance for pregnancy plan and early fetal treatment. Rare allelic mutations and structural variations can also be screened using cSMART primers, to expand pathogenic mutation spectrum for *MMACHC*. NIPT provides additional choice for high-risk pregnant women in alleviating anxiety and other contraindications caused by invasive operation. Moreover, the application of the cSMART assay could be expanded to genetic disorders with a high incidence, thus helping with disease prevention and treatment, especially for metabolic diseases with serious consequences. Besides, from cblC type MMA, there are many genetic disorders with a high incidence and hotspot mutations in specific population, such as c.1006C > T mutation in *CBS* gene mutation of homocystinuria in Qatar (El-Said et al., 2006; Al-Dewik et al., 2019), and c.394C > T mutation in *MMACHC* gene cobalamin C defect in North India (Kaur et al., 2021). Recently, a prenatal multigene screening assay called PreSeek covering more than 30 genes has been applied in clinical service (Zhang et al., 2019).

Unlike the common hybridization capture with the haplotype-based relative haplotype dosage method (Lo et al., 2010; Lam et al., 2012), direct sequencing methods, including the cSMART assay, cannot be used in cases with pseudogene interferences, such as in *SMN1* or *CYP21A2* directly (Ma et al., 2014; Parks et al., 2017). However, recently advanced technologies, such as long-read sequencing (LRS), can construct longer and more integrated haplotypes with more informative SNPs (Hui et al., 2017; Wenger et al., 2019). Targeted LRS may reduce cost close to current trio haplotyping methods. Importantly, it can construct pathogenic haplotypes no longer depending on the known affected proband. cSMART assay testing informative SNPs from targeted LRS of couples with RHDO analysis would provide a novel and precise proband-free NIPT way. Besides, the unique total read of cSMART that passed the QC was as ≥500, which is higher than the sequencing depth of current hybridization captured with haplotype-based relative haplotype dosage methods (200–400 ×) (Parks et al., 2017; Ye et al., 2018; Che et al., 2020; Han et al., 2020). More unique reads indicate less informative SNPs would be enough for future cSMART assay testing informative SNPs from targeted LRS of couples with RHDO analysis. This solves the technological limitation that the maximum primer number for cSMART, as a multiplex PCR-based assay, is less than hybridization probes in one reaction. The higher read number and accurate testing of hundreds of informative SNPs would allow reliable screening for dozens of common monogenic disorders in a single test.

By reviewing published NIPT studies for monogenic disorder based on cSMART so far, 291 cases were found involving seven autosomal recessive and three mitochondrial genes from multiple prenatal diagnosis centers (Table 2). The overall concordance rate is 95.88% (279/291), and the sensitivity and specificity are 95.77% (88.14–99.12%) and 97.27% (94.16–98.99%), respectively, indicating the high testing performance of the cSMART assay in various monogenic disorders. Method verification can be performed in laboratory-developed test laboratory, and the clinical approval certificate would be easily obtained following the rules of conventional multiplex PCR.

**TABLE 2:** A summary of the published cSMART applications of NIPT for different monogenic disorders.

Disease	Gene	Case numbers	Fetal genotype concordant rate	Sensitivity and specificity	Target allele (region) numbers	Fetal fraction and genotyping algorithm	Publication date
Wilson disease	*ATP7B*	4	100.00% (4/4)	100.00%, 100.00%	4 variants	No	2015 Jan Lv et al. (2015)
Inherited non-syndromic hearing loss	*GJB2*, *GJB3*, *SLC26A4*, *RNR1*, *TRNL1*, and *COX1*	25	100.00% (25/25)	100.00%, 100.00%	22 variants: *GJB2* (5), *GJB3* (5), *SLC26A4* (8), *RNR1* (2), *TRNL1* (1), and *COX1* (1)	No	2016 Dec Chen et al. (2016)
Autosomal recessive non-syndromic hearing loss	*GJB2* and *SLC26A4*	80	91.25% (73/80)	83.33%, 95.16%	39 variants: GJB2 (12) and *SLC26A4* (27)	FF	2017 Dec Han et al. (2017)
PKU	*PAH*	18	100.00% (18/18)	100.00%, 100.00%	16 pathogenic variants and 5 common polymorphisms	FF	2018 Feb Duan et al. (2018)
PKU	*PAH*	33	96.97% (32/33)	100.00%, 96.15%	All exons and intronic flanking regions	FF and MLE	2019 Nov Lv et al. (2019)
β-Thalassemia	*HBB*	102	97.06% (99/102)	100.00%, 97.26%	46 known Chinese *HBB* variants	FF and MLE	2021 Jan Lv et al. (2021)
cblC type MMA	*MMACHC*	29	96.55% (28/29)	100.00%, 100.00%	All coding exons and intronic flanking regions	FF and MLE	This article
Total	10 genes	291	95.88% (279/291)	95.77%, 97.27%			

All the data were original results from these studies with no quality control to remove low-quality cases. FF, fetal fraction; MLE, maximum likelihood estimation.

In this study, all the FFs were higher than 5% (range 8.63–17.37%), which may be due to the advanced gestational age (range 16–25 weeks). In the 10–20 weeks of the gestational period, FF was reported to be 10–15%, and 1–3% cases had an FF that was 4% (Canick et al., 2013). NIPT practice in aneuploidy detection suggests the cutoff value of FF to be larger than 4% and gestational age to be more than 12 weeks. Because the current version of cSMART also requires an FF of ≥5%, the recommended gestation age would also be more than 12 weeks, as similar as the application of regular NIPT in a clinical setting. However, the *P* value was generated using the MLE, which comprehensively considers the impact of sequencing reads and FF. Therefore, the *P* value could be taken as a unique key QC value and applied to cases with a lower FF but higher *P* value in the first trimester. If NIPT can be used stably in the earlier gestational period (10 weeks or less), before the clinical symptoms are observed by prenatal ultrasound examination, the cSMART assay would be more significant. At that time, more effective prenatal treatment can be offered in time, such as the administration of dexamethasone by the mother for fetuses at risk of virilizing congenital adrenal hyperplasia (Lajic et al., 2004).

## Conclusion

A novel NIPT assay covering the coding regions and proximal intronic regions of the cblC type MMA pathogenic gene *MMACHC* was developed. NIPT and IPD were performed, and the concordance rate achieved was 100.00% (27/27) after removing two QC-failed cases, indicating high sensitivity and specificity of the cSMART assay compared with IPD. In the future, advanced cSMART will be developed at an earlier testing gestational age with low fetal fraction; thus, pregnancy management and fetal treatment could be provided in the first trimester. Combined with long-read sequencing or other analysis algorithm, cSMART could also be used for NIPT for pseudogene, exon(s) or large genomic fragment deletion/duplication, and other complex conditions. Existing NIPT studies for monogenic disorders indicate that cSMART is accurate and universal, with high prospects for clinical application.

## Data Availability

According to national legislation/guidelines, specifically the Administrative Regulations of the People’s Republic of China on Human Genetic Resources (http://www.gov.cn/zhengce/content/2019-06/10/content_5398829.htm, http://english.www.gov.cn/policies/latest_releases/2019/06/10/content_281476708945462.htm), no additional raw data is available at this time. Data of this project can be accessed after an approval application to the Genome Sequence Archive in National Genomics Data Center, China National Center for Bioinformation/Beijing Institute of Genomics, Chinese Academy of Sciences (GSA-Human: HRA001625) that are publicly accessible at https://ngdc.cncb.ac.cn/gsa-human.
