# Supplementary material for: Noninvasive Prenatal Testing of Methylmalonic Acidemia cblC Type Using the cSMART Assay for MMACHC Gene Mutations
Source: Front Genet. 2022 Jan 7;12:750719. doi: 10.3389/fgene.2021.750719 (PMC8777107; doi:10.3389/fgene.2021.750719)
Supplement: Supplementary file 1 [file DataSheet3.docx]

**Supplementary material 3. The detailed deduction of the maternal-fetal genotypes.**

In order to simplify the statistical model principle of fetal genotype derivation, only explanation for parental heterozygous variation was described. Mutant allelic percent (alternative ratio, ALT ratio) and minor allelic ratio was described in **Table 1** in theory. Here, A (a) represent wild-type genotypes, B (b) represent mutant genotypes. Capital letters (A, B) for maternal/paternal alleles, lower letters (a, b) for fetal alleles.

**Table 1. Genotype of maternal-fetal allele in maternal plasma and its corresponding expected alternative and minor ratio.**

| **Genotype** | **Expected Alt ratio** | **Expected minor ratio** |
| --- | --- | --- |
| AAaa | 0 | 0 |
| BBbb | 0 | 0 |
| ABaa | 0.5-FF/2 | 0.5-FF/2 |
| ABab | 0.5 | 0.5 |
| AAab | FF/2 | FF/2 |
| ABbb | 0.5+FF/2 | 0.5-FF/2 |

ALT, alternative. Fetal fraction, FF.

Different maternal-fetal genotypes are digitally represented by minor ratio in the form of four genotypes including AAaa/BBbb, AAab/BBab, ABaa/ABbb, ABab. The genotypes and corresponding relationship for expected minor ratio and fetal fraction (FF) are listed in **Table 2**.

**Table 2. Genotypes and the expected minor ratio for four maternal-fetal genotypes**

| Genotype | AAaa/BBbb | AAab/BBab | ABaa/ABbb | ABab |
| --- | --- | --- | --- | --- |
| Expected minor ratio | 0 | FF/2 | 0.5-FF/2 | 0.5 |

**Figure 1. Binomial distributions of minor depth and their density distribution for four genotypes.**

The maximum likelihood estimation (MLE) algorithm based on binomial distribution method is applied to deduce maternal-fetal genotypes. Here, expected minor depth equals to total depth multiplied by expected minor ratio. For better visualization in the figure, total depth is assumed to be fixed and the same, and minor depth would represent the size of minor ratio. Minor depth and its probability distribution would be generated. However, because the value of FF is small (about 10%), the probability distribution of expected minor depth is also very narrow. The probability of a single event of expected minor depth is low, so the binomial distribution can be used to fit its distribution. Then, the mirror depth and its density distribution diagram under the binomial distribution model for four expected values was plotted in **Figure 1**.

In the following, approaching degree (D) between the real test minor depth (blue dotted lines) and expected minor ratio (red lines) in four genotypes are calculated (**Figure 1**). Four D values are generated (D_1_, D_2_, D_3_, D_4_), and the most probable genotype (G) was calculated by the maximum D value as following formula (up). The probability (P) of most probable genotype is deduced by the maximum D divided by sum of all approaching degree (D_1_, D_2_, D_3_ and D_4_).


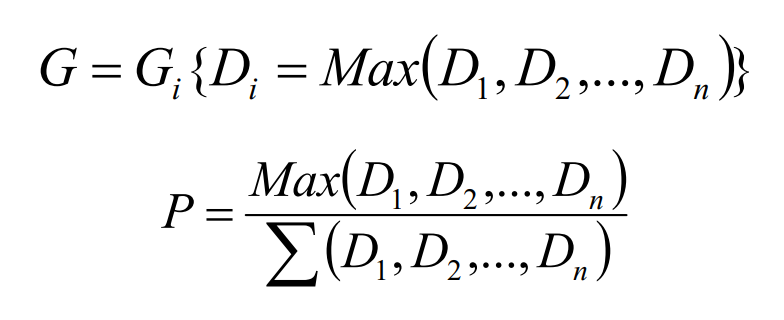


In real calculation, Expected Minor Ratio= Minor depth/Total depth, and it equals to observed mutation unique counts (m)/the total unique sequencing read (T) as describe in the main body.
