# Supplementary material for: Noninvasive Prenatal Testing of Methylmalonic Acidemia cblC Type Using the cSMART Assay for MMACHC Gene Mutations
Source: Front Genet. 2022 Jan 7;12:750719. doi: 10.3389/fgene.2021.750719 (PMC8777107; doi:10.3389/fgene.2021.750719)
Supplement: Supplementary file 2 [file DataSheet2.docx]

**Supplementary material 2. The deduction of fetal fraction**

The fetal fraction (FF) was calculated from the informative SNPs filtered from 76 selected heterozygous SNPs. The fetal allelic fraction (Δ) is half of FF. Specifically, FF was deduced from the change (Δ%) of the maternal allelic percentage (A) due to fetal inheritance alleles (A and B) for different maternal/fetal genotypes as follows:

AA/AB: A% = 100−Δ%;

AB/AA: A% = (50+ Δ%) and AB/BB: A% = (50−Δ) %;

BB/AB: A% = (0+Δ) %.

A two-step SNP filter was performed for the final deduction of FF. First, significantly different (p<0.001) SNP outliers was removed by a Chi-squared test from the set of 76 Δ% values for each sample. As for SNPs with borderline maternal/fetal genotypes, they are distinguished as informative or non-informative using maximum likelihood estimation (MLE), and informative SNPs were reserved for the following analysis. The FF equals to the double of median Δ% value of final reserved data set of informative SNPs.
