# Supplementary material for: Noninvasive Prenatal Testing of Methylmalonic Acidemia cblC Type Using the cSMART Assay for MMACHC Gene Mutations
Source: Front Genet. 2022 Jan 7;12:750719. doi: 10.3389/fgene.2021.750719 (PMC8777107; doi:10.3389/fgene.2021.750719)
Supplement: Supplementary file 3 [file DataSheet1.docx]

**Supplementary material 1**

**Contents list:**

**1) Supplementary Table 1. Primer sequence for targeting *MMACHC***

**2) Supplementary Table 2. 76 heterozygous SNP information**

**3) Supplementary Table 3. Invasive and noninvasive prenatal diagnosis results for 29 pregnancies at risk for cblC**

**4) Supplementary Figure 1. The distribution of primers in the upstream of exon 2 and the coverage area of amplification**

**5) Supplementary Table 4. NIPT results of two cSMART testing of PD0614 and PD0579**

**1) Supplementary Table 1. Primer sequence for targeting *MMACHC***

| **Primer set** | **Primer name** | **Primer sequence (from 3` to 5`)** |
| --- | --- | --- |
| Primer set 1 | MMACHCE1-1F | ATTCCCCAGCAAGCTCAGCG |
|  | MMACHCE1-1R | GAATGAAGTCTCAAGGACAATTGG |
|  | MMACHCE2-1F | AGCCTGGCCTGAACTTTCT |
|  | MMACHCE2-1R | CTGGAGAGGGCCAGTCTAG |
|  | MMACHCE2-3F | CCCAAACCTACAGCTGCCTC |
|  | MMACHCE2-3R | AGCTGGGGGAAAACTGAGC |
|  | MMACHCE3-1F | GACAGTACCCTCTATTTTGTCCACT |
|  | MMACHCE3-1R | TCAGCATGAGGGGAGTTATGAC |
|  | MMACHCE4-1F | AAGGGGACCTCCATGACCTTG |
|  | MMACHCE4-1R | CCAAGTCAACTGCCATCATTGC |
|  | MMACHCE4-3F | TCAGCCCTCAGAGAAGCCTAGT |
|  | MMACHCE4-3R | GCCCAATAGGGCCAATCGTTG |
| Primer set 2 | MMACHCE1-2F | ATGATTGGCTTCGTTGCAACT |
|  | MMACHCE1-2R | TTCGCCCTAGAACAGCAGGAG |
|  | MMACHCE2-2F | GCCCTCAAGCCCTTCTTG |
|  | MMACHCE2-2R | GTCAAACATGGCAGGCGT |
|  | MMACHCE3-2F | TAAAGGCCTCTCCCTACCAGG |
|  | MMACHCE3-2R | ACCAGTCTATCTCAGCCTCTAT |
|  | MMACHCE4-2F | ACCTCATGACTGTGTACCTACAAG |
|  | MMACHCE4-2R | TTCTGGGTGGCAGATCTGG |
|  | MMACHCE4-4F | GGTACTTGCTAGGACTTAATTGGC |
|  | MMACHCE4-4R | CCACCATAAATCAGGGTCCAC |

**2) Supplementary Table 2. 76 heterozygous SNP information**

| **No.** | **SNP** | **Chr** | **Position** | **Heterozygosis Freq in CHB-JPT** |
| --- | --- | --- | --- | --- |
| 1 | rs7549197 | chr.1 | 31681014 | 0.5 |
| 2 | rs4659766 | chr.1 | 237200616 | 0.5 |
| 3 | rs10177436 | chr.2 | 4925630 | 0.5 |
| 4 | rs11885000 | chr.2 | 42163072 | 0.5 |
| 5 | rs2024489 | chr.2 | 217661530 | 0.5 |
| 6 | rs6782081 | chr.3 | 159277496 | 0.5 |
| 7 | rs1498646 | chr.3 | 97749522 | 0.5 |
| 8 | rs1598856 | chr.4 | 103446115 | 0.5 |
| 9 | rs1905850 | chr.4 | 96434992 | 0.5 |
| 10 | rs2571468 | chr.4 | 30215673 | 0.5 |
| 11 | rs10065122 | chr.5 | 95610520 | 0.5 |
| 12 | rs10085169 | chr.5 | 121601488 | 0.5 |
| 13 | rs1011709 | chr.6 | 78813219 | 0.5 |
| 14 | rs1048394 | chr.6 | 158518507 | 0.5 |
| 15 | rs4607417 | chr.6 | 41978274 | 0.5 |
| 16 | rs10227916 | chr.7 | 1754509 | 0.5 |
| 17 | rs10259686 | chr.7 | 133633152 | 0.5 |
| 18 | rs2074337 | chr.7 | 27436685 | 0.5 |
| 19 | rs10097465 | chr.8 | 113510766 | 0.5 |
| 20 | rs10875448 | chr.8 | 141083615 | 0.5 |
| 21 | rs1157338 | chr.8 | 13696026 | 0.5 |
| 22 | rs4743716 | chr.9 | 99075707 | 0.5 |
| 23 | rs10904392 | chr.10 | 5068113 | 0.5 |
| 24 | rs10508351 | chr.10 | 8391429 | 0.5 |
| 25 | rs7075034 | chr.10 | 17547113 | 0.5 |
| 26 | rs697325 | chr.11 | 35635460 | 0.5 |
| 27 | rs728648 | chr.11 | 44019643 | 0.5 |
| 28 | rs10891936 | chr.11 | 116126460 | 0.5 |
| 29 | rs11047009 | chr.12 | 23776824 | 0.5 |
| 30 | rs11056390 | chr.12 | 15332867 | 0.5 |
| 31 | rs11612412 | chr.12 | 97422543 | 0.5 |
| 32 | rs2324776 | chr.13 | 65746182 | 0.5 |
| 33 | rs1164591 | chr.13 | 97089950 | 0.5 |
| 34 | rs1094506 | chr.13 | 81263389 | 0.5 |
| 35 | rs11620770 | chr.14 | 32700259 | 0.5 |
| 36 | rs182745 | chr.14 | 70443834 | 0.5 |
| 37 | rs12587148 | chr.14 | 33988502 | 0.5 |
| 38 | rs11637057 | chr.15 | 25719127 | 0.5 |
| 39 | rs11858322 | chr.15 | 26187841 | 0.5 |
| 40 | rs4261498 | chr.15 | 88175687 | 0.5 |
| 41 | rs1971791 | chr.15 | 70576138 | 0.5 |
| 42 | rs1070502 | chr.16 | 10265157 | 0.5 |
| 43 | rs7193727 | chr.16 | 83340836 | 0.5 |
| 44 | rs11651108 | chr.17 | 32448136 | 0.5 |
| 45 | rs1394386 | chr.17 | 31689590 | 0.5 |
| 46 | rs2279962 | chr.17 | 4688998 | 0.5 |
| 47 | rs10163625 | chr.18 | 21699237 | 0.5 |
| 48 | rs685435 | chr.18 | 34255303 | 0.5 |
| 49 | rs537063 | chr.18 | 34255116 | 0.5 |
| 50 | rs392332 | chr.19 | 33105743 | 0.5 |
| 51 | rs12610248 | chr.19 | 39501550 | 0.5 |
| 52 | rs1509116 | chr.20 | 8295535 | 0.5 |
| 53 | rs2867800 | chr.20 | 42874509 | 0.5 |
| 54 | rs1201915 | chr.20 | 59274805 | 0.5 |
| 55 | rs1513737 | chr.21 | 24166144 | 0.5 |
| 56 | rs2249508 | chr.21 | 38009239 | 0.5 |
| 57 | rs2834782 | chr.21 | 36500952 | 0.5 |
| 58 | rs2070369 | chr.21 | 36156012 | 0.5 |
| 59 | rs12627745 | chr.21 | 46797389 | 0.5 |
| 60 | rs7276777 | chr.21 | 36159252 | 0.5 |
| 61 | rs2834642 | chr.21 | 36186319 | 0.5 |
| 62 | rs2825928 | chr.21 | 21352994 | 0.5 |
| 63 | rs8133010 | chr.21 | 44126321 | 0.5 |
| 64 | rs2073382 | chr.21 | 47818298 | 0.5 |
| 65 | rs2831703 | chr.21 | 29702823 | 0.5 |
| 66 | rs2829505 | chr.21 | 26385726 | 0.5 |
| 67 | rs868092 | chr.21 | 43863521 | 0.5 |
| 68 | rs8133676 | chr.21 | 20075866 | 0.5 |
| 69 | rs2835804 | chr.21 | 38921767 | 0.5 |
| 70 | rs2223081 | chr.21 | 29778032 | 0.5 |
| 71 | rs9637299 | chr.21 | 44360107 | 0.5 |
| 72 | rs670316 | chr.21 | 40914124 | 0.5 |
| 73 | rs228039 | chr.21 | 44072193 | 0.5 |
| 74 | rs1035239 | chr.22 | 20793914 | 0.5 |
| 75 | rs6007500 | chr.22 | 45583935 | 0.5 |
| 76 | rs2001121 | chr.22 | 27118181 | 0.5 |

**3) Supplementary Table 3. Invasive and noninvasive prenatal diagnosis results for 29 pregnancies at risk for cblC**

| **Case No.** | **Maternal age (yrs)** | **GD** | **Mat genotype** | **Pat genotype** | **IPD result** | **NIPT by cSMART** | | | | | | | | | **Concordance of fetal genotype with IPD** |
| --- | --- | --- | --- | --- | --- | --- | --- | --- | --- | --- | --- | --- | --- | --- | --- |
|  |  |  |  |  |  | **Input DNA (ng)** | **Mutation site** | **Total reads** | **Mut reads** | **Mut Ratio** | **FF** | **Genotype**^#^ | ***P* value**^†^ | **QC^‡^** |  |
| PD0556 | 29 | 124 | c.609G>A/N | c.658_660delAAG/N | c.609G>A/c.658_660delAAG | 11.76 | c.609G>A | 536 | 280 | 52.24% | 11.74% | ABab | 70.88% | PASS | Concordant |
|  |  |  |  |  |  |  | c.658_660del | 520 | 40 | 7.69% | 11.74% | AAab | 100.00% |  |  |
| PD0580 | 33 | 115 | c.609G>A/N | c.658_660delAAG/N | c.609G>A/N | 22.34 | c.609G>A | 1126 | 547 | 48.58% | 12.30% | ABab | 99.03% | PASS | Concordant |
| PD0581 | 27 | 115 | c.80A>G/N | c.609G>A/N | c.80A>G/N | 22.33 | c.80A>G | 1394 | 679 | 48.71% | 8.96% | ABab | 91.59% | PASS | Concordant |
| PD0588 | 29 | 115 | c.482G>A/N | c.271dupA/N | c.271dupA/N | 12.18 | c.482G>A | 710 | 320 | 45.07% | 11.07% | ABaa | 96.78% | PASS | Concordant |
|  |  |  |  |  |  |  | c.271dupA | 730 | 44 | 6.03% | 11.07% | AAab | 100.00% |  |  |
| PD0614 | 29 | 113 | c.567dupT/N | c.567dupT/N | c.567dupT/N | 10.51 | c.567dupT | 828 | 398 | 48.07% | 8.63% | ABab | 58.12% | P value | Concordant |
| PD0625 | 31 | 134 | c.609G>A/N | c.427C>T/N | c.427C>T/N | 7.21 | c.609G>A | 959 | 437 | 45.57% | 10.00% | ABaa | 97.61% | PASS | Concordant |
|  |  |  |  |  |  |  | c.427C>T | 966 | 42 | 4.35% | 10.00% | AAab | 100.00% |  |  |
| PD0638 | 34 | 125 | c.609G>A/N | c.217C>T/N | c.609G>A/N | 6.82 | c.609G>A | 766 | 377 | 49.22% | 9.79% | ABab | 92.48% | PASS | Concordant |
| PD0640 | 31 | 125 | c.609G>A/N | c.658_660delAAG/N | c.658_660delAAG/N | 23.52 | c.609G>A | 1104 | 513 | 46.47% | 8.78% | ABaa | 93.06% | PASS | Concordant |
|  |  |  |  |  |  |  | c.658_660del | 1023 | 26 | 2.54% | 8.78% | AAab | 100.00% |  |  |
| PD0644 | 27 | 135 | c.609G>A/N | c.276+1G>A/N | c.609G>A/N | 13.44 | c.609G>A | 700 | 339 | 48.43% | 12.73% | ABab | 94.81% | PASS | Concordant |
| PD0645 | 31 | 122 | c.609G>A/N | c.658_660delAAG/N | c.609G>A/c.658_660delAAG | 14.73 | c.609G>A | 896 | 458 | 51.12% | 12.06% | ABab | 98.43% | PASS | Concordant |
|  |  |  |  |  |  |  | c.658_660del | 822 | 50 | 6.08% | 12.06% | AAab | 100.00% |  |  |
| PD0646 | 32 | 138 | c.609G>A/N | c.658_660delAAG/N | c.609G>A/N | 27.72 | c.609G>A | 1458 | 776 | 53.22% | 17.37% | ABab | 99.71% | PASS | Concordant |
| PD0660 | 25 | 118 | c.609G>A/N | c.394C>T/N | c.609G>A/c.394C>T | 15.12 | c.609G>A | 825 | 424 | 51.39% | 9.09% | ABab | 78.98% | PASS | Concordant |
|  |  |  |  |  |  |  | c.394C>T | 598 | 14 | 2.34% | 9.09% | AAab | 100.00% |  |  |
| PD0661 | 34 | 112 | c.658_660delAAG/N | c.609G>A/N | c.658_660delAAG/c.609G>A | 9.24 | c.658_660del | 657 | 319 | 48.55% | 9.49% | ABab | 76.20% | PASS | Concordant |
|  |  |  |  |  |  |  | c.609G>A | 657 | 43 | 0.0654 | 0.0949 | AAab | 100.00% |  |  |
| PD0662 | 32 | 115 | c.609G>A/N | c.658_660delAAG/N | c.658_660delAAG/N | 34.86 | c.609G>A | 1675 | 706 | 42.15% | 12.00% | ABaa | 100.00% | PASS | Concordant |
|  |  |  |  |  |  |  | c.658_660del | 1754 | 116 | 6.61% | 12.00% | AAab | 100.00% |  |  |
| PD0666 | 28 | 120 | c.658_660delAAG/N | c.217C>T/N | c.217C>T/N | 7.74 | c.658_660del | 541 | 238 | 43.99% | 12.21% | ABaa | 98.04% | PASS | Concordant |
|  |  |  |  |  |  |  | c.217C>T | 866 | 50 | 5.77% | 12.21% | AAab | 100.00% |  |  |
| PD0673 | 28 | 127 | c.609G>A/N | c.609G>A/N | c.609G>A/c.609G>A | 13.86 | c.609G>A | 906 | 490 | 54.08% | 8.78% | ABbb | 95.29% | PASS | Concordant |
| PD0674 | 28 | 120 | c.609G>A/N | c.609G>A/N | N/N | 17.64 | c.609G>A | 1266 | 568 | 44.87% | 10.00% | ABaa | 99.87% | PASS | Concordant |
| PD0679 | 29 | 128 | c.609G>A/N | c.658_660delAAG/N | c.658_660delAAG/N | 24.78 | c.609G>A | 1099 | 483 | 43.95% | 12.42% | ABaa | 99.97% | PASS | Concordant |
|  |  |  |  |  |  |  | c.658_660del | 1020 | 49 | 4.80% | 12.42% | AAab | 100.00% |  |  |
| PD0680 | 38 | 120 | c.609G>A/N | c.609G>A/N | N/N | 7.14 | c.609G>A | 861 | 394 | 45.76% | 8.67% | ABaa | 95.68% | PASS | Concordant |
| PD0687 | 29 | 124 | c.609G>A/N | c.217C>T/N | c.217C>T/N | 6.81 | c.609G>A | 989 | 429 | 43.38% | 11.95% | ABaa | 99.98% | PASS | Concordant |
|  |  |  |  |  |  |  | c.217C>T | 1349 | 70 | 5.19% | 11.95% | AAab | 100.00% |  |  |
| PD0688 | 27 | 130 | c.609G>A/N | c.482G>A/N | c.609G>A/N | 24.36 | c.609G>A | 993 | 477 | 48.04% | 9.64% | ABab | 70.35% | PASS | Concordant |
| PD0698 | 31 | 128 | c.609G>A/N | c.80A>G/N | c.80A>G/N | 11.34 | c.609G>A | 842 | 339 | 40.26% | 11.62% | ABaa | 100.00% | PASS | Concordant |
|  |  |  |  |  |  |  | c.80A>G | 894 | 31 | 3.47% | 11.62% | AAab | 100.00% |  |  |
| PD0700 | 37 | 124 | c.658_660delAAG/N | c.394C>T/N | N/N | 13.02 | c.658_660del | 867 | 356 | 41.06% | 15.10% | ABaa | 100.00% | PASS | Concordant |
| PD0701 | 35 | 180 | c.609G>A/N | c.658_660delAAG/N | c.658_660delAAG/N | 8.43 | c.609G>A | 759 | 324 | 42.69% | 12.52% | ABaa | 99.97% | PASS | Concordant |
|  |  |  |  |  |  |  | c.658_660del | 642 | 39 | 6.07% | 12.52% | AAab | 100.00% |  |  |
| PD0709 | 30 | 112 | c.609G>A/N | c.567dupT/N | c.609G>A/N | 9.24 | c.609G>A | 609 | 308 | 50.57% | 15.65% | ABab | 99.84% | PASS | Concordant |
| PD0675 | 32 | 132 | c.482G>A/N | c.609G>A/N | c.482G>A/c.609G>A | 22.26 | c.482G>A | 809 | 386 | 47.71% | 9.63% | ABab | 99.98% | PASS | Concordant |
|  |  |  |  |  |  |  | c.609G>A | 554 | 22 | 3.97% | 9.63% | AAab | 100.00% |  |  |
| PD0616 | 30 | 123 | c.606_641delinsCTT/N | c.658_660delAAG/N | N/N | 19.74 | c.606_641delinsCTT | 720 | 231 | 32.08% | 10.50% | ABaa | 100.00% | PASS | Concordant |
| PD0579 | 33 | 121 | c.609G>A/N | c.609G>A/N | c.609G>A/N | 14.02 | c.609G>A | 486 | 228 | 46.91% | 9.17% | ABaa | 66.96% | READS | Discordant |
| PD0639 | 29 | 132 | c.609G>A/N | c.609G>A/N | c.609G>A/N | 20.16 | c.609G>A | 1026 | 502 | 48.93% | 9.28% | ABab | 91.58% | PASS | Concordant |

Abbreviations: GD, gestational days; Mat, maternal; Pat, paternal; IPD, invasive prenatal diagnosis; NIPT, noninvasive prenatal testing; cSMART, circulating single molecule and resequencing technology; Mut, mutation; FF, fetal fraction; *P* value, probability value by maximum likelihood.

^#^ The maternal-fetal genotype was deduced using the maximum likelihood algorithm (refer to **Supplementary material 3** and manuscript reference Jiang et al., 2012).

^†^ The formula for calculating probability of the most likely maternal-fetal genotype has been previously described (refer to **Supplementary material 2** and manuscript reference Jiang et al., 2012; Song et al., 2016).

^‡^ The cut-off to call a fetal genotype was a FF ≥ 5%, cSMART reads ≥ 500 and *P* value ≥ 70%.


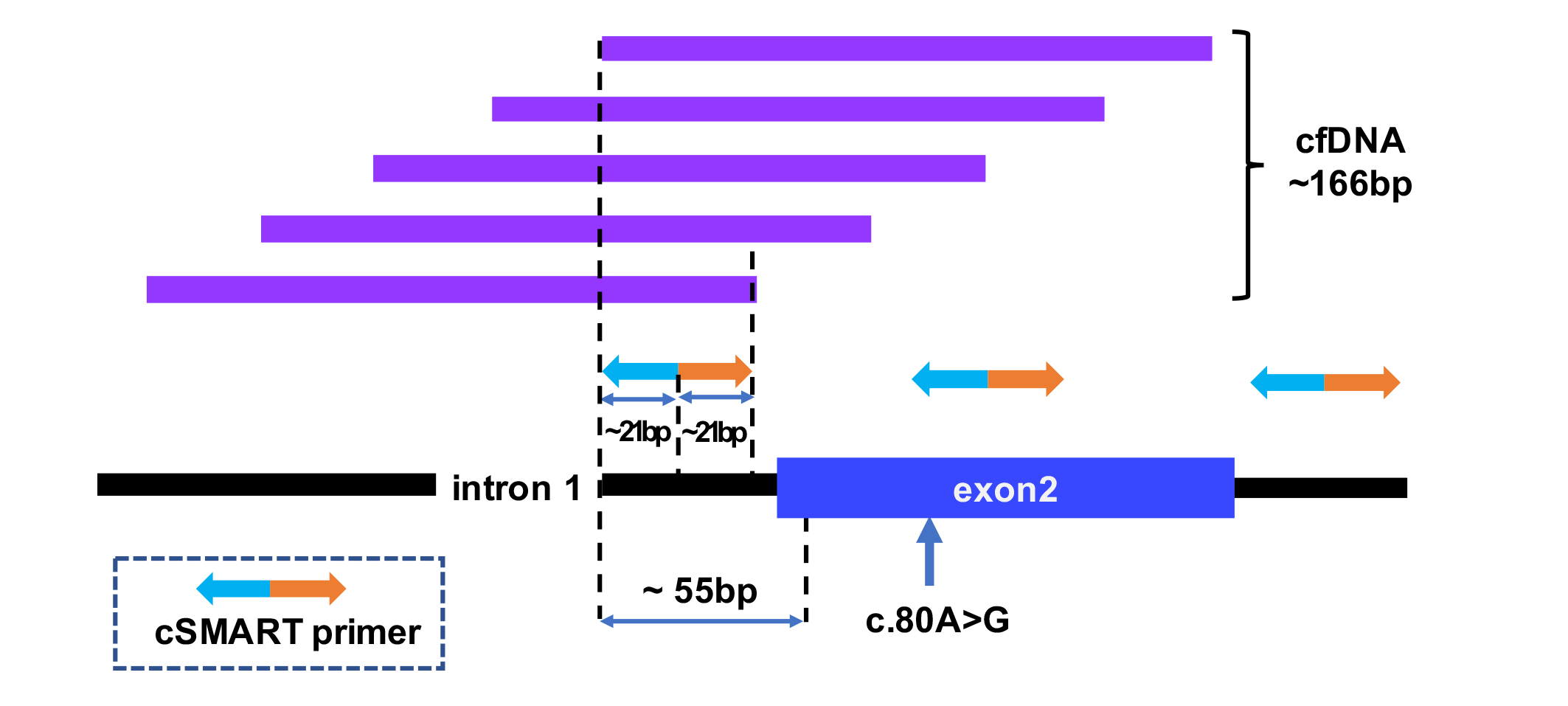


**Supplementary Figure 1. The distribution of primers in the upstream of exon 2 and the coverage area of amplification.** The primers can cover the 124bp (166bp - 21bp × 2) sequence upstream and downstream of the primers, and 179bp (55bp + 166bp - 21bp × 2) sequence in deep intron region.

**Supplementary Table 4. NIPT results of two cSMART testing of PD0614 and PD0579**

| Case No. | Testing | Mat genotype | Total reads | Mut reads | Mut Ratio | FF | Genotype | *P* value |
| --- | --- | --- | --- | --- | --- | --- | --- | --- |
| PD0614 | The first testing | c.567dupT | 828 | 398 | 48.07% | 8.63% | ABab | 58.12% |
|  | The second testing | c.567dupT | 572 | 271 | 47.38% | 9.28% | ABaa | 57.90% |
| PD0579 | The first testing | c.609G>A | 486 | 228 | 46.91% | 9.17% | ABaa | 66.96% |
|  | The second testing | c.609G>A | 429 | 198 | 46.15% | 9.11% | ABaa | 77.33% |

Abbreviations: Mat, maternal; cSMART, circulating single molecule and resequencing technology; Mut, mutation; FF, fetal fraction; *P* value, probability value by maximum likelihood.
